# Efficacy of dialectical behavior therapy for adolescent self-harm and suicidal ideation: a systematic review and meta-analysis

**DOI:** 10.1017/S0033291721001355

**Published:** 2021-05

**Authors:** Oswald D. Kothgassner, Andreas Goreis, Kealagh Robinson, Mercedes M. Huscsava, Christian Schmahl, Paul L. Plener

**Affiliations:** 1 Department of Child and Adolescent Psychiatry, Medical University of Vienna, Vienna, Austria; 2 Department of Clinical and Health Psychology, Faculty of Psychology, University of Vienna, Vienna, Austria; 3 Outpatient Unit for Research, Teaching and Practice, Faculty of Psychology, University of Vienna, Vienna, Austria; 4 School of Psychology, Te Herenga Waka – Victoria University of Wellington, Wellington, New Zealand; 5 Department of Psychosomatic Medicine and Psychotherapy, Central Institute of Mental Health, Medical Faculty Mannheim, Heidelberg University, Mannheim, Germany; 6 Department of Child and Adolescent Psychiatry and Psychotherapy, University of Ulm, Ulm, Germany

**Keywords:** Adolescence, borderline personality disorder, DBT-A, self-harm, self-injury, suicidal ideation

## Abstract

**Background:**

Given the widespread nature and clinical consequences of self-harm and suicidal ideation among adolescents, establishing the efficacy of developmentally appropriate treatments that reduce both self-harm and suicidal ideation in the context of broader adolescent psychopathology is critical.

**Methods:**

We conducted a systematic review and meta-analysis of the Dialectical Behaviour Therapy for Adolescents (DBT-A) literature on treating self-injury in adolescents (12–19 years). We searched for eligible trials and treatment evaluations published prior to July 2020 in MEDLINE/PubMed, Scopus, Google Scholar, EMBASE, and the Cochrane Library databases for clinical trials. Twenty-one studies were identified [five randomized-controlled trials (RCTs), three controlled clinical trials (CCTs), and 13 pre-post evaluations]. We extracted data for predefined primary (self-harm, suicidal ideation) and secondary outcomes (borderline personality symptoms; BPD) and calculated treatment effects for RCTs/CCTs and pre-post evaluations. This meta-analysis was pre-registered with OSF: osf.io/v83e7.

**Results:**

Overall, the studies comprised 1673 adolescents. Compared to control groups, DBT-A showed small to moderate effects for reducing self-harm (*g* = −0.44; 95% CI −0.81 to −0.07) and suicidal ideation (*g* = −0.31, 95% CI −0.52 to −0.09). Pre-post evaluations suggested large effects for all outcomes (self-harm: *g* = −0.98, 95% CI −1.15 to −0.81; suicidal ideation: *g* = −1.16, 95% CI −1.51 to −0.80; BPD symptoms: *g* = −0.97, 95% CI −1.31 to −0.63).

**Conclusions:**

DBT-A appears to be a valuable treatment in reducing both adolescent self-harm and suicidal ideation. However, evidence that DBT-A reduces BPD symptoms was only found in pre-post evaluations.

Self-injury, suicidal ideation, and suicide attempts represent major mental health concerns for adolescents around the world. Suicide is the leading cause of death for female adolescents and the third highest cause of death for male adolescents in the western world (Collaboration GBoDP, 2016; Hawton, Saunders, & O'Connor, 2012). In addition, meta-analyses estimate that 22.9% of adolescents have engaged in self-harm whereby they deliberately and directly damage their body tissue in the absence of suicidal ideation (Gillies et al., 2018). Although there is ongoing debate about the nature of the relationship between self-harm and suicidal thoughts and behaviors (Hamza, Stewart, & Willoughby, 2012), the two are related (Gillies et al., 2018). Meta-analyses of longitudinal studies estimate that people who engage in self-harm have 4.27 greater odds of subsequently attempting suicide, and those who engage in deliberate self-harm – regardless of suicidal intent – have 1.51 greater odds of subsequent death by suicide (Ribeiro et al., 2016).

Adolescence represents a key developmental period for both self-harm and suicidality (Wyman, 2014). Although suicide is uncommon before the age of 15, the prevalence of suicide strongly increases from late adolescence to early adulthood (Bertolote & Fleischmann, 2002). Self-harm also tends to begin during early adolescence around 13–15 years, with growing evidence to suggest that earlier onset of self-harm increases the risk of a more severe trajectory (Ammerman, Jacobucci, Kleiman, Uyeji, & McCloskey, 2018; Groschwitz et al., 2015; Plener, Schumacher, Munz, & Groschwitz, 2015). Self-harm and suicidal ideation typically present alongside other psychiatric disorders, such as affective and stress-associated disorders (Nock, Joiner, Gordon, Lloyd-Richardson, & Prinstein, 2006), and among adults the most well-established link is with borderline personality disorder (BPD; Ferrara et al., 2012; Kaplan et al., 2016). Given the widespread nature and clinical consequences of adolescent self-injury, establishing developmentally appropriate treatments that reduce both self-harm and suicidal ideation in the context of broader adolescent psychopathology is critical.

One treatment which has received growing interest is Dialectical Behaviour Therapy for Adolescents (DBT-A). DBT was initially developed to treat women diagnosed with BPD at high-risk for suicide (e.g. Chapman, 2006; Linehan, Heard, & Armstrong, 1993) and is widely recommended as an established therapeutic approach for people with BPD, particularly when reducing self-injury is a priority (APA, 2006; National Collaborating Center for Mental Health, 2009). Subsequently, a dialectical behavioral approach was adapted for adolescents, which prioritizes self-harm and suicidal ideation as the primary targets for therapeutic intervention (Miller, Rathus, & Linehan, 2017; Rathus & Miller, 2002). DBT-A is a manualized treatment approach intended for outpatient settings comprised of weekly individual therapy with concurrent participation in a skills-group and which includes parental participation. In particular, DBT-A focuses on developing mindfulness, distress tolerance, interpersonal effectiveness, and emotion regulation behavioral skills as the main therapeutic tools for overcoming pervasive emotion dysregulation and suicidal ideation (Miller et al., 2017; Rathus & Miller, 2015).

To date, two reviews evaluating the efficacy of psychosocial treatments for reducing adolescent self-harm and suicidal ideation have highlighted DBT-A as a promising treatment (Glenn, Franklin, & Nock, 2015; Kothgassner, Robinson, Goreis, Ougrin, & Plener, 2020). However, previous meta-analysis focused exclusively on a small number (*k* = 3) of randomized controlled trials (RCTs; Kothgassner et al., 2020), and the systematic review of controlled clinical trials (CCTs, trials including a control group, but which lack randomization) and pre-post evaluations only included studies published prior to July 2013 (*k* = 5, no RCTs; Glenn et al., 2015). Given both the clinical importance of responding effectively to adolescent self-harm and suicidal ideation, and the limited number of DBT-A RCTs highlighted in previous reviews, we decided to include all studies across different stages of clinical evaluation in order to provide the most comprehensive synthesis of the current evidence. In addition, although DBT has shown success in treating BPD symptoms in adults, the efficacy of DBT-A for treating BPD symptoms among adolescents who self-injure remains to be evaluated (Cristea et al., 2017). Thus, in this review, we include RCTs, CCTs, and pre-post evaluation studies to evaluate the efficacy of DBT-A for reducing self-harm, suicidal ideation and BPD symptoms among adolescents, and conduct subgroup analyses to compare the results for RCTs with those of less rigorous studies. The greater heterogeneity in studies also allows us to assess whether characteristics of the study (e.g. participant age, treatment duration) moderate the meta-analytic effect of DBT-A on outcomes, in order to better understand the parameters under which DBT-A is most successful.

## Method

### Search strategy and inclusion criteria

We conducted a search of MEDLINE/PubMed, Scopus, Google Scholar, EMBASE, and the Cochrane Library databases for clinical trials for studies published from the beginning of database records until 31 July 2020 using the keywords ‘Dialectical Behaviour Therapy’ OR ‘DBT-A’ and combinations of the keywords ‘Self-harm’ OR ‘Self-Injury’, ‘Suicidal Ideation’, OR ‘Suicide’ with an age limitation. Studies were included in the meta-analysis if they reported an RCT or CCT comparing DBT-A with a control intervention or a pre-post evaluation of DBT-A and reported outcomes for self-harm and/or suicidal ideation in adolescents aged 12–19 who had engaged in self-injury at least once. We also excluded studies focusing solely on pharmacological treatments. No limitations on language or publication status were invoked, and no other inclusion or exclusion criteria were applied.

We analyzed the frequency of self-harm episodes and suicidal ideation as primary outcome measures, with BPD symptoms as a secondary outcome measure. The title, abstract, and main text of each study were examined, with the exclusion of documents occurring at each stage. The initial search generated 932 results. Title and abstracts were screened for eligibility and full-text papers were obtained where necessary to evaluate inclusion. After screening, 21 studies – all peer-reviewed journal articles in English – were identified and included in our meta-analysis.

### Data extraction and analysis

Data from included studies were entered into a spreadsheet independently by two authors (ODK and KR). A third author (AG) reviewed and discussed differences until consensus was reached. We coded the sample and intervention characteristics of each study included in the meta-analysis. For analyses of the efficacy of DBT-A in RCTs and CCTs, the primary outcome was the standardized mean difference (Hedges' *g*) between the DBT-A and control interventions on self-harm and suicidal ideation measured post-intervention. The secondary outcome was the standardized mean difference (Hedges' *g*) for BPD symptoms in the DBT-A and control interventions measured post-intervention. For analyses regarding pre-post treatment effects, we computed the standardized mean difference (Hedges' *g*) based on means and standard deviations (Dunlap et al., 1996) before and after DBT-A intervention using the formula *g* = (*M*_post_-*M*_pre_)/SD_pooled_, where *M*_post_ is the mean of the measure after the intervention and *M*_pre_ the mean before the intervention, with SD_pooled_ as the standard deviation for both measurements, defined as SD_pooled_ = SQRT(SD_pre_^2^ + SD_post_^2^)/2 (Lakens, 2013).

Means, standard deviations, and sample sizes were retrieved and inserted into a spreadsheet. If means or standard deviations were not reported in studies or Supplemental materials, conversions via Revman Calculator (Cochrane Collaboration, 2014) or formulas (Card, 2012) were conducted. If self-harm episodes were reported as proportions or odds ratios, they were transformed to Hedges' *g* via the formula provided in Lipsey and Wilson (2001). Effect size calculations and meta-analyses were conducted with the metafor package for R (Viechtbauer, 2010). Following established conventions, an effect size of 0.20 was considered a small effect size, 0.50 a medium effect, and 0.80 a large effect size (Cohen, 1988). Random-effects models were applied to estimate aggregated effect sizes. Heterogeneity across study outcomes was reported with *I*^2^ values, where 25% indicates low heterogeneity, 50% moderate, and 75% high heterogeneity (Higgins, Thompson, Deeks, & Altman, 2003). Moderator analyses (meta-regression) were conducted to test whether treatment duration, gender composition, and participant mean age moderated the effect of the DBT-A on each outcome. Egger's regressions were conducted to estimate publication bias (Sterne & Egger, 2005), with adjusted effect sizes calculated using trim-and-fill analyses and, based on funnel plot asymmetry, numbers of imputed missing studies (Duval & Tweedie, 2000). All data and analysis code are available on the Open Science Framework (doi:10.17605/OSF.IO/YZXPJ).

### Risk of bias assessment

Risk of bias for each study was assessed using predefined criteria based on the Agency for Healthcare Research and Quality method guide (see Supplement 1; Viswanathan et al., 2018). Each study was assessed in regard to randomization, selection and attrition bias, confounding bias, measurement bias, and statistical problems and received a rating of low, moderate or high risk of bias. Low risk of bias indicates that the study was judged to be valid, moderate risk indicates concerns that probably do not invalidate the study's results, and high risk of bias indicates significant concerns that likely invalidate the study's results. Two investigators (KR and ODK) independently assessed the studies and differences were reviewed until consensus was reached.

## Results

**Fig. 1.**
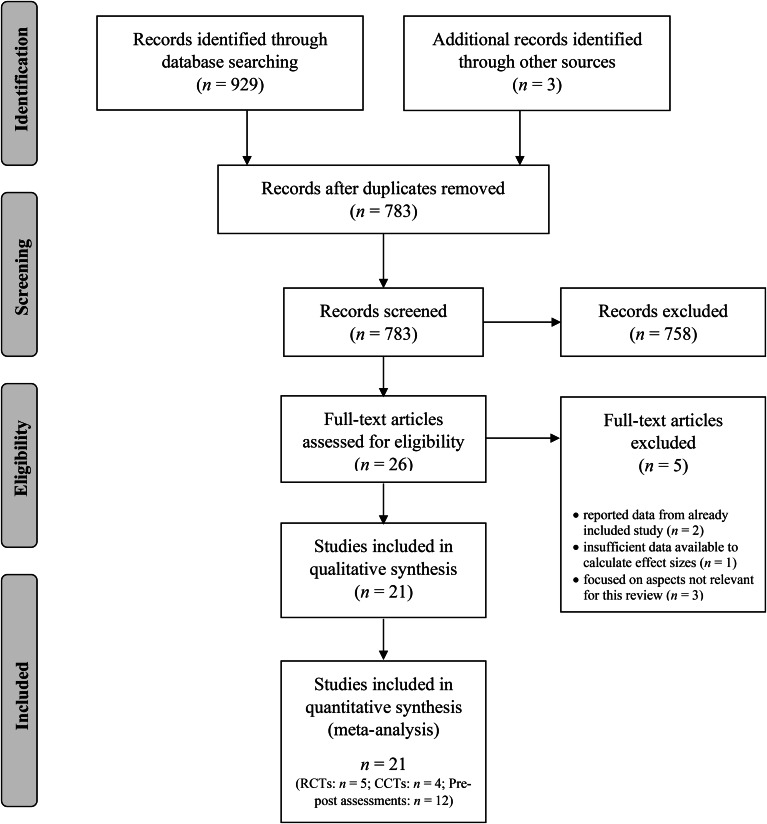
PRISMA flowchart showing the screening, exclusion, and inclusion criteria.

In total, 21 studies were identified (see Fig. 1 for the PRISMA flow diagram). Five studies were RCTs, three studies were CCTs, and 13 were pre-post evaluation studies (see Table 1 for an overview of study characteristics). The study by Rathus and Miller (2002) – originally included as a CCT – was included as a pre-post evaluation study, given that data for the control intervention was unavailable. In total, the 21 studies comprised 1673 adolescents. Overall, 1063 participants received DBT-A interventions, and 610 received control interventions. A sufficient number of studies (*k* > 1; Pigott, 2012) were identified to calculate aggregate effect sizes for self-harm and suicidal ideation outcomes in RCTs, CCTs, and pre-post evaluations. However, only one controlled study reported BPD symptoms as an outcome, and so this outcome was solely assessed among pre-post evaluations. Across studies, participants tended to be female (*M* = 82%) and 15.4 years old (s.d. = 1.3). An average of 7% of participants dropped-out across studies (range: 0–40%) and 63% received concurrent psychopharmacological intervention over the course of the intervention (*k* = 11 studies did not provide sufficient data about medication).

**Table 1.** Study characteristics

Study Year	Study design	Sample size	Country	Control intervention	% Female	Age range (mean)	Treatment duration in months	Setting	Eligibility criteria	Outcome (measures)	% Drop-out
Apsche, Bass, and Siv (2006)	RCT	20	USA	Mode deactivation therapy	0%	15–18 (DBT-A: 15.9, Control: 16.1)	12	Residential treatment	Not stated	SI (SIQ)	0%
Berk, Starace, Black, and Avina (2020)	Pre-post	22	USA	–	92%	12–17 (15.2)	6	Outpatient	Recent history of suicidal and/or self-injury behaviors	SH (past 6-month frequency), SI (SIQ/SIQ-Jr), BPD (SCID-II)	8%
Buerger et al. (2019)	Pre-post	72	Germany	–	92%	12–17 (15.7)	6.25	Outpatient	3 + BPD criteria; fluency in German	SH and SI (SITBI-G), BPD (LPI)	18%
Courtney and Flament (2015)	Pre-post	42	Canada	–	93%	Adolescents 15 and above (16.5)	3	Outpatient	SIQ score >30, or self-injury in the past 4 months	SH (chart review) BPD (LPI)	51%
Fischer and Peterson (2015)	Pre-post	7	USA	–	100%	14–17 (16.2)	6	Outpatient	Binge eating within past 4 weeks; 1+ suicide attempt or episode of self-injury within past year; height and weight within or above typical limits for age and stage of development	SH (DSHI past month)	30%
Fleischhaker et al. (2011)	Pre-post	10	Germany	–	100%	13–19 (not stated)	6	Outpatient	SH and/or suicidal behavior within past 16 weeks; BPD diagnosis or 3+ BPD criteria	SH (LPC)	25%
Geddes, Dziurawiec, and Lee (2013)	Pre-post	6	Australia	–	100%	14 years 6 month–15 years 1 month (15.1)	6.5	Outpatient	Average cognitive ability and established reading level; self-injury and/or suicidal ideation within past 12 months; 3+ BPD criteria	SH and SI (in-house questionnaire)	33%
Gillespie, Joyce, Flynn, and Corcoran (2019)	Pre-post	84	Ireland	–	85%	13–18 (15.7)	4	Outpatient	Demonstration of emotional and behavioral dysregulation; persistent self-injury with 1+ episodes of self-injury or suicidal acts within the past 16 weeks or chronic suicidal ideation; expressed commitment by both adolescent and parent/guardian	SH (review of diary cards), SI (in-house questionnaire), BPD (BSL-23)	16%
Goldstein, Axelson, Birmaher, and Brent (2007)	Pre-post	10	USA	–	80%	14–18 (15.8)	6	Outpatient	Bipolar diagnosis with an acute manic, mixed, or depressive episode within past 3 months; engaged in a pharmacotherapy regimen; 1+ parent/guardian willing to participate in family sessions	SH (K-SADS), SI (MSSI)	10%
Goldstein et al. (2015)	RCT	20	USA	Treatment-as-usual	75%	12–18 (DBT: 15.8, Control: 16.8)	12	Outpatient	Bipolar disorder diagnosis; an acute manic, mixed, or depressive episode within past 3 months; willingness to engage in pharmacotherapy; 1+ parent/guardian willing to participate in family sessions	SH (LIFE), SI (SIQ-Jr)	2%
James, Taylor, Winmill, and Alfoadari (2008)	Pre-post	16	UK	–	100%	15–18 (16.4)	12	Outpatient	History of >6 months severe and persistent self-injury	SH (clinical interview), BPD (SCID-II)	13%
James, Winmill, Anderson, and Alfoadari (2011)	Pre-post	18	UK	–	88%	13–17 (15.5)	3	Outpatient	History of >6 months persistent self-injury	SH (clinical interview), BPD (SCID-II)	28%
Katz et al. (2004)	CCT	53	Canada	Treatment-as-usual	84%	14–17 (15.4)	0.5	Inpatient	Admitted to inpatient unit following a suicide attempt or with severe suicidal ideation; agreed to stay in the hospital for brief treatment	SH (LPC), SI (SIQ-Jr)	9%
McCauley et al. (2018)	RCT	173	USA	Individual and group supportive therapy	94%	12–18 (14.9)	6	ED, inpatient, outpatient services, and community services	1+ lifetime suicide attempt; elevated past-month suicidal ideation (⩾24 on the SIQ-Jr); ⩾3 lifetime self-injury episodes, including 1 episode in the 12 weeks before screening, 3+ BPD criteria	SH (SASII), SI (SIQ-Jr), BPD (SCID-II)	40%
McDonell et al. (2010)	CCT	155	USA	Historical control	58%	12–17 (15.54)	12	Inpatient	Not stated	SH (quality assurance database)	0%
Mehlum et al. (2014)	RCT	77	Norway	Enhanced usual care	88%	12–18 (DBT: 15.9, Control: 15.3)	4.75	Outpatient	2+ self-injury episodes, with 1+ within the last 16 weeks; 2+ BPD criteria (and the self-destructive criterion), or, 1+ criterion of BPD and at least 2 subthreshold-level criteria; fluency in Norwegian	SH (frequency count), SI (SIQ-Jr), BPD (BSL-23)	0%
Perepletchikova et al. (2011)	Pre-post	11	USA	–	55%	8–11 years 6 months (9.83)	1.5	Elementary school	Not stated	SI (MFQ)	0%
Rathus and Miller (2002)	CCT	13	USA	Treatment-as-usual	93%	Range not stated (DBT: 16.1, Control: 15.0)	3	Outpatient	A suicide attempt with last 16 weeks or current suicidal ideation; a BPD diagnosis or 3+ BPD criteria	SI (SSI), BPD (LPI)	Defers by group TAU: 60%, DBT-A: 38%
Santamarina-Perez et al. (2020)	RCT	35	Spain	Treatment-as-usual + group therapy	89%	12–17 years 11 months (DBT: 15.3, Control: 15.2)	4	Outpatient	Repetitive SH (proposed DSM-V criteria) and/or suicide attempts over the last 12 months and at current high risk of suicide; 1+ parent/guardian willing to participate in family sessions	SH (medical records), SI (SIQ-Jr)	20%
Tebbett-Mock et al. (2020)	CCT	801	USA	Historical control	66%	12–17 (DBT): 15.7 (1.4) Control: 15.6 (1.5)	0.36	Inpatient	Not stated	SH (medical records), SI (observation hours for SI)	Not reported
Woodberry and Popenoe (2008)	Pre-post	28	USA	–	82%	13–18 (16.0)	3.75	Inpatient, outpatient, community services, and schools	History of suicide attempts, self-injury, and/or intense and unstable affect or relationships within the past 3–6 months; adolescent and parent/guardian willing to commit to the 15-week treatment	SH and SI (TSCC)	37%

Outcomes: BPD, borderline personality disorder; SH, self-harm; SI, suicidal ideation. Measures: BSL-23, Borderline Symptom List; DSHI, Deliberate Self-Harm Inventory; K-SADS, Schedule for Affective Disorders and Schizophrenia for School-Age Children; LIFE, Longitudinal Interval Follow-Up Evaluation Self-Injurious/Suicidal Behaviour Scale; LPC, Lifetime Parasuicide Count; LPI, The Life Problems Inventory; MFQ, Mood and Feeling Questionnaire; MSSI, Modified Scale for Suicidal Ideation; SASII, Suicide Attempt Self-Injury Interview; SCID-II, Structured Clinical Interview for DSM-IV Axis II; SITBI-G, Self-Injurious Thoughts and Behaviours Interview (German version); SIQ, Suicidal Ideation Questionnaire; SIQ-Jr, Suicidal Ideation Questionnaire Junior; SSI, Scale for Suicidal Ideation; TSCC, Trauma Symptom Checklist for Children.

**Fig. 2.**
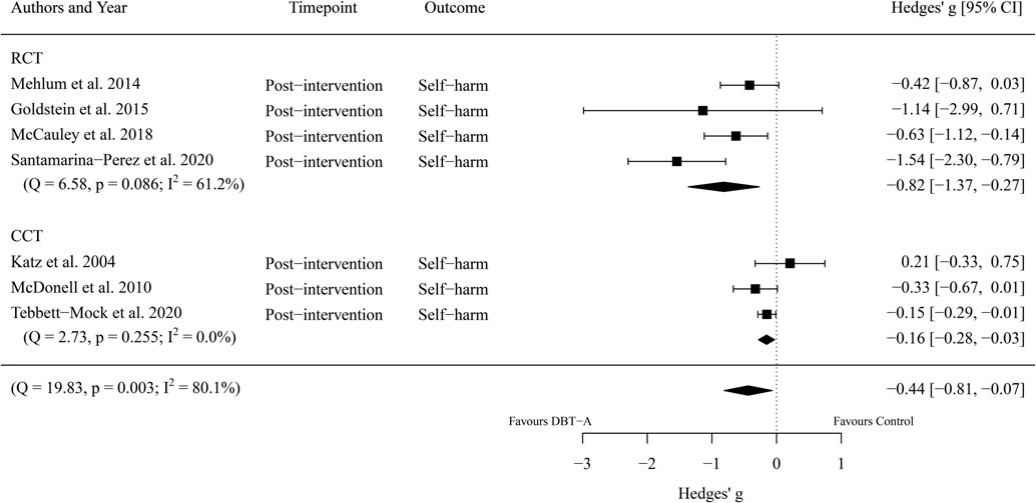
Forest plot of trials comparing the effect of DBT-A and controls on symptoms of self-harm.

First, we considered the overall efficacy of DBT-A, compared to control interventions, for reducing adolescent self-harm. Seven effect sizes encompassing 1314 participants (DBT-A: *n* = 714, Control: *n* = 600, *k* = 7) were extracted. Meta-analysis revealed a significant difference between groups (*g* = −0.44, 95% CI −0.81 to −0.07, *p* = 0.021) with a high heterogeneity between studies (*I*^2^ = 80.13%). That is, DBT-A interventions showed a small-to-medium improvement in reducing self-harm compared to control interventions. See Fig. 2 for a comparison of DBT-A (relative to control interventions) by study type (RCT or CCT) for reducing self-harm.

**Fig. 3.**
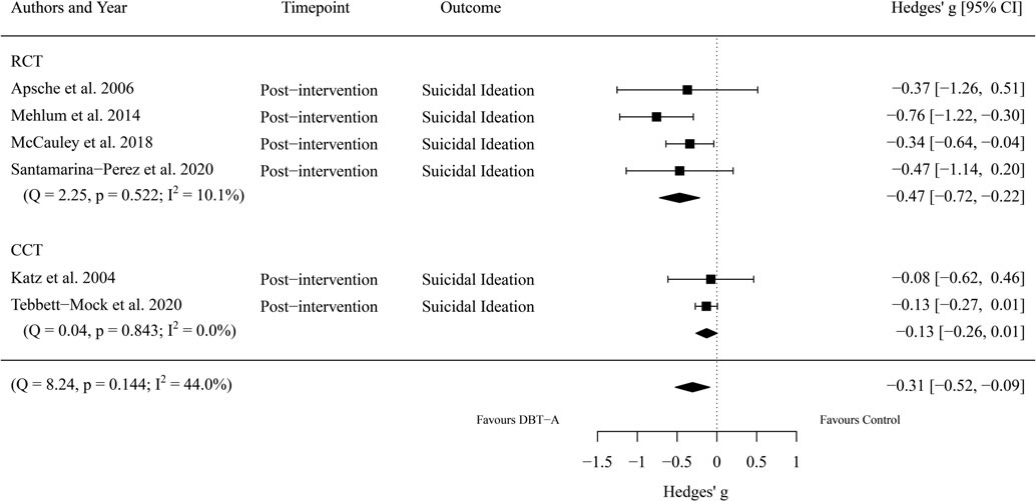
Forest plot of trials comparing the effect of DBT-A and controls on symptoms of suicidal ideation.

Next, we considered the overall efficacy of DBT-A, compared to control interventions, for reducing adolescent suicidal ideation. Five effect sizes encompassing 1159 participants (DBT-A: *n* = 604, Control: *n* = 555, *k* = 6) were extracted. Meta-analysis revealed a significant difference between groups (*g* = −0.31, 95% CI −0.52 to −0.09, *p* = 0.006), with moderate heterogeneity between studies (*I*^2^ = 44.05%). That is, DBT-A was moderately more effective at reducing suicidal ideation than control interventions. See Fig. 3 for a comparison of DBT-A (relative to control interventions) by study type (RCT or CCT) for reducing suicidal ideation.

In terms of BPD symptoms, Mehlum et al. (2014) conducted the only RCT which assessed the efficacy of DBT-A in reducing BPD symptoms. They reported that both DBT-A (*n* = 39) and ‘enhanced usual care’ (non-manualized standard care provided at least once weekly for the purpose of the trial; *n* = 38) reduced BPD symptoms, but found no significant group difference. Given that only one RCT was eligible for inclusion in this review, we did not conduct meta-analysis of the effect of DBT-A and control interventions on BPD symptoms.

Moving beyond group comparisons of treatment and control interventions, we next considered within-subject changes in self-harm, suicidal ideation, and BPD symptoms following DBT-A intervention. Table 2 shows the effects of DBT-A across outcome measures. Among participants who received DBT-A, pre-post comparisons indicate large reductions in self-harm (*g* = −0.98), suicidal ideation (*g* = −1.16), and BPD symptoms (*g* = −0.97). All effects were statistically significant, with suicidal ideation (*I*^2^ = 54.58) and BPD symptoms (*I*^2^ = 43.51) showing moderate heterogeneity, and self-harm showing low-to-moderate heterogeneity (*I*^2^ = 0.00) across studies.

**Table 2.** Pre-post treatment effects (Hedges' *g*) and heterogeneity indices of DBT-A

Outcome	*k*	*n*	Hedges' *g*	95% CI	*I*^2^
Reduction at treatment completion					
Self-harm	16	498	−0.98*	−1.15 to −0.81	0.00
Suicidal ideation	11	299	−1.16*	−1.51 to −0.80	54.58
Borderline personality disorder symptoms	5	218	−0.97*	−1.31 to −0.63	43.51

Hedges' *g* indicates change from pre to post-intervention such that a negative effect size indicates a reduction in that outcome following DBT-A.

* Indicates effect sizes that are statistically significant (*p* < 0.001).

Next, in order to better understand the parameters in which DBT-A is most effective, we assessed whether study characteristics moderated the efficacy of DBT-A in reducing adolescent self-harm and suicidal ideation. We conducted multiple meta-regression to assess whether treatment duration (in months), age (in years), or proportion of young women in the overall sample (compared to young men) moderated the size of meta-analytic effects between DBT-A and control interventions (see Supplement 2 for all coefficients). Treatment duration was negatively associated with the change in effect sizes for suicidal ideation (*b* = −0.08, *p* = 0.012), but not self-harm (*b* = −0.06, *p* = 0.379). That is, a longer duration of DBT-A led to a larger reduction in suicidal ideation when compared to control interventions. In contrast, gender composition and age of the sample did not influence the effect size of the difference between DBT-A and control interventions for self-harm or suicide ideation (all *p*s > 0.05).

We repeated these meta-regressions for the assessments of effect sizes in pre-post evaluations (see Supplement 2 for all coefficients). Again, treatment duration was negatively associated with larger effect sizes for reducing BPD symptoms (*b* = −0.29, *p* = 0.016), such that the longer the DBT-A treatment the larger the reduction in symptoms from pre to post. No other moderating effects of treatment duration were found. Similarly, gender composition and age of the sample did not influence any of the outcomes in pre-post analyses.

We next consider the potential impact of publication bias in the studies examined in these meta-analyses. For each outcome (both in the effect as compared to control interventions and in pre-post comparisons), visual inspection of funnel plots suggested that across all four outcomes, studies were symmetrically distributed. That is, we found no evidence for publication bias in the studies assessing self-harm, suicidal ideation, or BPD symptoms included in the present meta-analyses. In addition, Egger's regression found no evidence for funnel plot asymmetry in the analyses we conducted (all *z*s < 1.64, all *p*s > 0.100). Given that no indication of publication bias was found, no adjustments according to trim-and-fill analysis were conducted in any of the analyses.

Finally, we evaluate the quality of the studies included in the current review using predefined criteria based on the Agency for Healthcare Research and Quality method guide (Viswanathan et al., 2018). Overall, statistical problems were common with 57.1% of studies rated at ‘High’ risk of bias due to low sample sizes. High risk of measurement bias (38.1%) and confounding bias (33.3%) was also common, due to reliance on unvalidated instruments or inadequately addressing potential confounds. Taken together, quality assessments highlight the need for widespread adoption of standardized measurement instruments and well-powered replication studies (see Supplement 1).

## Discussion

Given the scarcity of studies investigating the efficacy of DBT-A in reducing adolescent self-harm and suicidal ideation, particularly in the context of comorbid psychopathology such as BPD, we conducted a systematic review of controlled trials to inform best-practice clinical decision making. Our meta-analysis included 21 studies comprised of 1673 participants and provides evidence to support the efficacy of DBT-A (compared to control interventions) for reducing self-harm and suicidal ideation as primary outcomes. The effect size for self-harm reduction in favor of DBT-A was large for RCTs and small-to-medium when CCTs were included. The effect sizes for suicidal ideation reduction in favor of DBT-A were small-to-medium for both RCTs and when all controlled studies were included in the analysis. The current review identified an insufficient number of studies to evaluate the efficacy of DBT-A in BPD symptoms.

A growing body of research demonstrates that therapeutic interventions for self-harm and suicidal ideation in general show limited efficacy (Fox et al., 2020; Kothgassner et al., 2020), highlighting the importance of isolating specific therapies which show promise for further development. In a review of all published RCTs targeted at reducing suicidal thoughts and behaviors, DBT showed a small treatment effect for self-harm, but had no effect on suicidal ideation (Fox et al., 2020). Similarly, in a review of controlled trials investigating the efficacy of DBT among adult samples found a small effect in favor of DBT for reducing self-injury, but no effect on suicidal ideation (DeCou, Comtois, & Landes, 2019). Focusing specifically on children and adolescents, our results reveal promising effects of DBT-A for both self-harm and suicidal ideation for both RCTs and CCTs. Given that adolescence is a key developmental period for both self-harm and suicidal ideation (Wyman, 2014), future research is needed to understand the underlying mechanism(s) of how DBT-A works to improve self-harm and suicidal ideation.

Across all studies, our findings indicate that longer duration of DBT-A may be crucial for greater efficacy, particularly for suicidal ideation. Additionally, longer treatment duration was associated with greater reductions in BPD symptoms in pre-post evaluations. These findings correspond to the DBT-A treatment hierarchy in which further BPD symptoms are addressed in later stages of therapy, after an initial focus on establishing sufficient behavior control. Since the therapeutic relationship can be considered as a critical reinforcement for people with BPD (Bedics, Atkins, Harned, & Linehan, 2015), a longer duration may mean a more effective use of the therapeutic relationship in terms of contingency management (Miller et al., 2017).

We also found larger effect sizes for self-harm and suicidal ideation in RCTs than in CCTs. This difference might be explained by the fact that these study types differ by recruitment setting. RCTs predominantly recruited adolescents receiving outpatient care (Goldstein et al., Mehlum et al., 2014; Santamarina-Perez et al., 2020), except for McCauley et al. (2018) who included adolescents recruited from both inpatient and outpatient settings, whereas all CCTs reporting self-harm and suicidal ideation outcomes consisted of participants recruited from inpatient settings (Katz, Cox, Gunasekara, & Miller, 2004; McDonell et al., 2010; Tebbett-Mock, Saito, McGee, Woloszyn, & Venuti, 2020). Another potential explanation may lie in the different methodological quality of RCT and CCT studies.

No study accounted for the combined effect of pharmacological treatment with DBT-A, despite the fact that psychopharmacological treatment for adolescents with BPD in general is common (Cailhol et al., 2013), and over half of participants treated in the included trials received additional psychopharmacological treatment. Further, some studies reported reduction of medication or adherence as an outcome variable (Katz et al., 2004; McDonell et al., 2010; Tebbett-Mock et al., 2020). However, to date the efficacy of a combined therapy approach remains unclear.

Critically, DBT-A targets both the adolescent and their family. Typically, adolescents continue to live in the environment where they acquired their dysfunctional patterns and so families are integrated into therapy in order to address invalidating behaviors within the family context. In terms of contingencies, this holistic approach reinforces skills and helps to decrease maladaptive behaviors by addressing both the adolescent's and parent's behavioral and communicative repertoire. Preliminary research provides tentative support for these mechanisms of change. In a non-randomized pilot of DBT-A among a small sample of ethnic minority adolescents, adaptive coping at pretreatment predicted subsequent increased use of DBT skills at post-treatment (Yeo et al., 2020). Secondary analysis of McCauley et al. (2018) revealed that adolescents who reported higher emotion dysregulation at baseline, and whose parents reported greater psychopathology and emotion dysregulation demonstrated greater reduction in self-harm following 6-months of DBT-A treatment (Adrian et al., 2019). In a longitudinal study of adults with a recent suicide attempt who received DBT treatment, participants with higher problem-focused coping and poorer access to emotion regulation strategies were more likely to reattempt suicide over the course of 2 years (Kuehn, King, Linehan, & Harned, 2020). However, future research is needed to better establish mechanisms of therapeutic change, as well as identify who stands to benefit most from DBT-A.

Our review has several limitations. First, only one RCT (Mehlum et al., 2014) assessed the impact of DBT-A (compared to control interventions) on BPD symptoms, preventing us from meta-analytically considering change in BPD symptoms as a secondary outcome. Second, we reported a moderately high heterogeneity among studies assessing the efficacy of DBT-A on self-harm. This heterogeneity might be due to the use of different control interventions, with some studies using specific psychotherapeutic control interventions (McCauley et al., 2018) and providing enhanced usual care or additional propositions (Mehlum et al., 2014; Santamarina-Perez et al., 2020). Alternatively, this heterogeneity may reflect differences in how self-harm was assessed; 58.8% (*k* = 7) of studies used unvalidated clinical interviews, medical records, daily diary cards, or instruments developed in-house to assess self-harm. Moreover, our meta-analysis is limited by the low number of effects available to be included which contributes to the wide confidence intervals of some of the estimates. Finally, young women made up 75% or more of the sample in most studies (81.0%, *k* = 17) included in this review. Thus, questions remain regarding the efficacy of DBT-A in reducing self-harm and suicidal ideation among young men and gender-diverse young people.

Despite these limitations, this review provides the most comprehensive analysis to date of the available evidence for the efficacy of DBT-A in reducing self-harm and suicidal ideation among adolescents. Current evidence indicates that DBT-A is superior to control interventions in reducing both self-harm and suicidal ideation among adolescents, with limited evidence of efficacy for reducing BPD symptoms. Future research should focus on improving the quality of evidence for the efficacy of DBT-A for underrepresented populations such as young men and gender diverse people, and for reducing BPD symptoms among adolescents in general. Greater investigation is also needed to understand the combined effect of DBT-A and psychopharmacological interventions, as well as to establish the feasibility and efficacy of DBT-A in teletherapeutic contexts in order to improve accessibility for young people who require specialized care for reducing their self-harm behavior and suicidal ideation.

## Supplementary material

For supplementary material accompanying this paper visit https://doi.org/10.1017/S0033291721001355. click here to view supplementary material
