# Supplementary material for: Efficacy of dialectical behavior therapy for adolescent self-harm and suicidal ideation: a systematic review and meta-analysis
Source: Psychol Med. 2021 Apr 20;51(7):1057–67. doi: 10.1017/S0033291721001355 (PMC8188531; doi:10.1017/S0033291721001355)
Supplement: Supplementary file 1 [file S0033291721001355sup.zip › S0033291721001355sup001.docx]

**Supplement 1**

Supplementary Table 1 shows Risk of Bias Assessments–predefined criteria for rating. In Table 2, we present the 21 studies’ assessment of quality sorted by design (Randomized Controlled Trials; RCTs, Clinical Controlled Trials; CCTs, pre-post design).

**Supplementary 1, Table 1: Risk of Bias Assessment – Categories for rating (adapted from Viswanathan et al., 2018)**

| **Randomization Bias** |  |
| --- | --- |
| Method of Randomization* | **Low**: Computer generated random allocation  **Moderate**: Other methods identified to randomize  **High**: Inadequate method of randomization (e.g., alternating), randomization approach cannot be determined or did not randomize |
| **Selection Bias** |  |
| Attrition Bias | **Low:** Low attrition or low differential loss  **Moderate:** Moderate attrition (20-39%) or moderate differential loss (5-29%)  **High:** High Attrition (≥ 40%) or high differential loss (≥ 30%), or cannot be determined |
| Creation of Comparable  Groups* | **Low:** No significant baseline differences among groups regarding primary outcome measures and inclusion/exclusion criteria  **Moderate:** Few baseline differences among groups  **High**: Multiple differences among groups, or does not state any baseline comparisons between groups |
| **Confounding Bias** |  |
| Control of confounding | **Low:** Addressed through study design (e.g., RCT) and/or statistical analysis  **Moderate:** Attempt made to control confounding, but doesn’t address all relevant confounders  **High:** No attempt to control confounders |
| **Measurement Bias** |  |
| Outcome Measurement | **Low:** Existing and validated measure used for self-harm and/or suicide ideation symptom severity  **Moderate:** Validated measurement for self-harm and /or suicide ideation symptom severity  **High:** Used originally developed instrument/items without validation |
| **Statistical Problems** | **Low:** Power-calculation, sufficient participants, reporting *p*-values and effect size estimates, using appropriate statistical methods  **Moderate:** Reporting *p*-values and effect size estimates, appropriate statistical methods  **High:** Small group sizes, insufficient data, or inappropriate statistical methods used |

* Indicator used only for RCTs

**Supplementary 1, Table 2: Risk of Bias Assessments**

|  | **Study** | **Randomization Bias** | **Selection Bias** | **Confounding Bias** | **Measurement Bias** | **Statistical Problems** |
| --- | --- | --- | --- | --- | --- | --- |
| **RCTs** | Apsche et al. (2006) | High | Low | Moderate | Low | High |
|  | Goldstein et al. (2015) | Moderate | Low | Moderate | Low | Moderate |
|  | McCauley et al. (2018) | Low | Moderate | Low | Low | Low |
|  | Mehlum et al. (2014) | Moderate | Low | Low | Moderate | Low |
|  | Santamarina-Perez et al. (2020) | Low | Moderate | Low | Moderate | Moderate |
|  |  |  |  |  |  |  |
| **CCTs** | Katz et al. (2004) | - | Low | Low | Moderate | Low |
|  | McDonell et al. (2010) | - | Low | High | High | High |
|  | Rathus & Miller (2002) | - | Low | High | Low | High |
|  | Tebbett-Mock et al. (2020) | - | Low | High | High | Moderate |
|  |  |  |  |  |  |  |
| **Pre-Post Trials** | Berk et al. (2019) | - | Low | Low | High | High |
|  | Buerger et al. (2019) | - | Low | Low | Low | Low |
|  | Courtney & Flament (2015) | - | High | Low | Moderate | Low |
|  | Firscher et al. (2014) | - | Moderate | High | Low | High |
|  | Fleischhaker et al. (2011) | - | Moderate | High | Low | High |
|  | Geddes et al. (2013) | - | Moderate | Moderate | High | High |
|  | Gillepie et al. (2019) | - | Low | Low | High | High |
|  | Goldstein et al. (2007) | - | Low | Moderate | Low | High |
|  | James et al. (2008) | - | Low | High | High | High |
|  | James et al. (2011) | - | Moderate | High | High | High |
|  | Perepletchikova et al. (2011) | - | Low | Moderate | High | High |
|  | Woodberry & Popenoe (2008) | - | Moderate | Low | Low | Low |

**References**

Viswanathan, M., Patnode, C. D., Berkman, N. D., Bass, E. B., Chang, S., Hartling, L., ... & Kane, R. L. (2018). Recommendations for assessing the risk of bias in systematic reviews of health-care interventions. *Journal of Clinical Epidemiology, 97*, 26–34. doi:10.1016/j.jclinepi.2017.12.004

**References included in meta-analysis**

*Apsche, J. A., Bass, C. K., & Siv, A. (2006). A treatment study of Mode Deactivation Therapy in an out patient community setting. *International Journal of Behavioral Consultation and Therapy*, *2*(2), 277-285. <http://dx.doi.org/10.1037/h0100782>.

*Berk, M. S., Starace, N. K., Black, V. P., & Avina, C. (2020). Implementation of dialectical behavior therapy with suicidal and self-harming adolescents in a community clinic. *Archives of suicide research*, *24*(1), 64-81. <https://doi.org/10.1080/13811118.2018.1509750>

*Buerger, A., Fischer-Waldschmidt, G., Hammerle, F., Auer, K. V., Parzer, P., & Kaess, M. (2019). Differential change of borderline personality disorder traits during dialectical behavior therapy for adolescents. *Journal of personality disorders*, *33*(1), 119-134. <https://doi.org/10.1521/pedi_2018_32_334>

*Courtney, D. B., & Flament, M. F. (2015). Adapted dialectical behavior therapy for adolescents with self-injurious thoughts and behaviors. *The Journal of Nervous and Mental Disease*, *203*(7), 537-544. <https://doi:10.1097/NMD.0000000000000324>.

*Fischer, S., & Peterson, C. (2015). Dialectical behavior therapy for adolescent binge eating, purging, suicidal behavior, and non-suicidal self-injury: A pilot study. *Psychotherapy*, *52*(1), 78-92. [https://doi.org/10.1037/a0036065](https://psycnet.apa.org/doi/10.1037/a0036065)

*Fleischhaker, C., Böhme, R., Sixt, B., Brück, C., Schneider, C., & Schulz, E. (2011). Dialectical behavioral therapy for adolescents (DBT-A): a clinical trial for patients with suicidal and self-injurious behavior and borderline symptoms with a one-year follow-up. *Child and adolescent psychiatry and mental health, 5*(1), 3. <https://doi.org/10.1186/1753-2000-5-3>

*Geddes, K., Dziurawiec, S., & Lee, C. W. (2013). Dialectical behaviour therapy for the treatment of emotion dysregulation and trauma symptoms in self-injurious and suicidal adolescent females: A pilot programme within a community-based child and adolescent mental health service. *Psychiatry journal*, *2013*. <https://doi.org/10.1155/2013/145219>

*Gillespie, C., Joyce, M., Flynn, D., & Corcoran, P. (2019). Dialectical behaviour therapy for adolescents: a comparison of 16‐week and 24‐week programmes delivered in a public community setting. *Child and Adolescent Mental Health*, *24*(3), 266-273. <https://doi.org/10.1111/camh.12325>

*Goldstein, T. R., Axelson, D. A., Birmaher, B., & Brent, D. A. (2007). Dialectical behavior therapy for adolescents with bipolar disorder: a 1-year open trial. *Journal of the American Academy of Child & Adolescent Psychiatry*, *46*(7), 820-830. <https://doi.org/10.1097/chi.0b013e31805c1613>

*Goldstein, T. R., Fersch-Podrat, R. K., Rivera, M., Axelson, D. A., Merranko, J., Yu, H., ... & Birmaher, B. (2015). Dialectical behavior therapy for adolescents with bipolar disorder: results from a pilot randomized trial. *Journal of child and adolescent psychopharmacology*, *25*(2), 140-149. <https://doi.org/10.1089/cap.2013.0145>

*James, A. C., Taylor, A., Winmill, L., & Alfoadari, K. (2008). A preliminary community study of dialectical behaviour therapy (DBT) with adolescent females demonstrating persistent, deliberate self‐harm (DSH). *Child and Adolescent Mental Health*, *13*(3), 148-152. <https://doi.org/10.1111/j.1475-3588.2007.00470.x>

*James, A. C., Winmill, L., Anderson, C., & Alfoadari, K. (2011). A preliminary study of an extension of a community dialectic behaviour therapy (DBT) programme to adolescents in the looked after care system. *Child and Adolescent Mental Health*, *16*(1), 9-13. <https://doi.org/10.1111/j.1475-3588.2010.00571.x>

*Katz, L. Y., Cox, B. J., Gunasekara, S., & Miller, A. L. (2004). Feasibility of dialectical behavior therapy for suicidal adolescent inpatients. *Journal of the American Academy of Child & Adolescent Psychiatry*, *43*(3), 276-282. <https://doi.org/10.1097/00004583-200403000-00008>

*McCauley, E., Berk, M. S., Asarnow, J. R., Adrian, M., Cohen, J., Korslund, K., ... & Linehan, M. M. (2018). Efficacy of dialectical behavior therapy for adolescents at high risk for suicide: a randomized clinical trial. *JAMA psychiatry*, *75*(8), 777-785. <https://doi:10.1001/jamapsychiatry.2018.1109>

*McDonell, M. G., Tarantino, J., Dubose, A. P., Matestic, P., Steinmetz, K., Galbreath, H., & McClellan, J. M. (2010). A pilot evaluation of dialectical behavioural therapy in adolescent long‐term inpatient care. *Child and Adolescent Mental Health*, *15*(4), 193-196. <https://doi.org/10.1111/j.1475-3588.2010.00569.x>

*Mehlum, L., Tørmoen, A. J., Ramberg, M., Haga, E., Diep, L. M., Laberg, S., ... & Grøholt, B. (2014). Dialectical behavior therapy for adolescents with repeated suicidal and self-harming behavior: a randomized trial. *Journal of the American Academy of child & adolescent psychiatry*, *53*(10), 1082-1091. <https://doi.org/10.1016/j.jaac.2014.07.003>

*Perepletchikova, F., Axelrod, S. R., Kaufman, J., Rounsaville, B. J., Douglas‐Palumberi, H., & Miller, A. L. (2011). Adapting dialectical behaviour therapy for children: Towards a new research agenda for paediatric suicidal and non‐suicidal self‐injurious behaviours. *Child and adolescent mental health*, *16*(2), 116-121. <https://doi.org/10.1111/j.1475-3588.2010.00583.x>

*Rathus, J.H., Miller, A.L. (2002). Dialectical Behavior Therapy Adapted for Suicidal Adolescents. *Suicide and Life-Threatening Behavior, 32*(2), 146-157.

*Santamarina‐Perez, P., Mendez, I., Singh, M. K., Berk, M., Picado, M., Font, E., ... & Cosi, A. (2020). Adapted dialectical behavior therapy for adolescents with a high risk of suicide in a community clinic: a pragmatic randomized controlled trial. *Suicide and Life‐Threatening Behavior*. <https://doi.org/10.1111/sltb.12612>

*Tebbett-Mock, A. A., Saito, E., McGee, M., Woloszyn, P., & Venuti, M. (2020). Efficacy of dialectical behavior therapy versus treatment as usual for acute-care inpatient adolescents. *Journal of the American Academy of Child & Adolescent Psychiatry*, *59*(1), 149-156. <https://doi.org/10.1016/j.jaac.2019.01.020>

*Woodberry, K. A., & Popenoe, E. J. (2008). Implementing dialectical behavior therapy with adolescents and their families in a community outpatient clinic. *Cognitive and behavioral practice*, *15*(3), 277-286. <https://doi.org/10.1016/j.cbpra.2007.08.004>
