# Supplementary material for: Efficacy of dialectical behavior therapy for adolescent self-harm and suicidal ideation: a systematic review and meta-analysis
Source: Psychol Med. 2021 Apr 20;51(7):1057–67. doi: 10.1017/S0033291721001355 (PMC8188531; doi:10.1017/S0033291721001355)
Supplement: Supplementary file 1 [file S0033291721001355sup.zip › S0033291721001355sup002.docx]

**Supplement 2**

**Supplementary 2, Table 1***.* Parameters of mixed-effects meta-regression on the efficacy of DBT-A in self-harm, suicidal ideation, and BPD symptoms.

|  | **RCTs and CCTs** | | | | | | **All studies pre to post** | | | | | | | | |
| --- | --- | --- | --- | --- | --- | --- | --- | --- | --- | --- | --- | --- | --- | --- | --- |
| **Predictors** | **Self-harm (*k =* 7)** | | | **Suicidal Ideation (*k =* 6)** | | | **Self-harm (*k =* 13)** | | | **Suicidal Ideation (*k =* 10)** | | | **Borderline Personality Disorder Symptoms (*k* = 5)** | | |
|  | *b* | *SE* | *p* | *b* | *SE* | *p* | *b* | *SE* | *p* | *b* | *SE* | *p* | *b* | *SE* | *p* |
| Treatment Duration (Months) | -0.06 | 0.07 | 0.379 | **-0**.**08** | **0**.**03** | **0**.**012** | -0.07 | 0.05 | 0.192 | -0.01 | 0.09 | 0.945 | **-0**.**29** | **0**.**12** | **0**.**016** |
| % Females | -2.00 | 2.86 | 0.484 | -1.47 | 0.76 | 0.055 | -0.01 | 0.01 | 0.208 | -0.01 | 0.01 | 0.781 | -0.01 | 0.01 | 0.226 |
| Age | -0.04 | 1.36 | 0.980 | -0.88 | 0.49 | 0.075 | 0.40 | 0.27 | 0.142 | -0.15 | 0.76 | 0.840 | – | – | – |
| *Q* | 1.31 | | | 7.26 | | | 4.28 | | | 0.45 | | | 5.90 | | |

Note. *k =* number of studies, *b* = unstandardized regression coefficient, SE = standard error of unstandardized regression coefficient. Due to the low number of original studies, age was not analysed as a predictor for BPD symptoms for RCTs and CCTs.** p < 0*.*05*
